# Robust Augmented-Diffusion Filtered-x Maximum Correntropy Criterion for Distributed Active Noise Control Under Impulsive Noise

**DOI:** 10.3390/e28101102

**Published:** 2026-10-10

**Authors:** Dongming Peng, Yigang He, Qianqian Liu

**Affiliations:** 1 School of Electrical Engineering and Automation, Hefei University of Technology, Hefei 230009, China; dmpeng_hfut@126.com; 2 School of Electrical Engineering, Wuhan University, Wuhan 430072, China; 3 School of Electrical Information Engineering, Jiangsu University of Technology, Changzhou 213001, China; liuqianqian@jsut.edu.cn

**Keywords:** distributed active noise control, augmented diffusion, impulsive noise, maximum correntropy criterion, robust adaptive filtering

## Abstract

Distributed multichannel active noise control (ANC) is scalable, but filtered-x least-mean-square updates are highly sensitive to impulsive disturbances. This paper proposes a robust augmented-diffusion filtered-x maximum correntropy criterion algorithm (R-ADFxMCC). It keeps the fixed admissible combination of augmented diffusion and replaces the local squared-error update with a normalized correntropy-gated recursion, whose kernel width follows a median-absolute-deviation estimate of the local residual scale. The resulting influence function is bounded and redescending. Under the stated independence and linearization approximations, the analysis gives a combination-weight-independent mean stability condition and a local steady-state excess mean-square error approximation; a symmetry argument proves zero steady-state mean bias without requiring independence between the gate and the regressors. In 30-realization simulations with one validation protocol for all step sizes, the median steady-state gains over augmented-diffusion FxLMS are 25.79, 21.84, 13.80 and 6.46 dB for single-tone, multitone, narrowband and broadband references under localized Bernoulli–Gaussian impulses. The augmented-diffusion M-estimate algorithm reaches the same steady state within 0.45 dB at the validation-selected step sizes, but on single-tone and narrowband references, R-ADFxMCC remains stable at step sizes at which the M-estimate algorithm diverges. Tests with α-stable noise, reverberation, secondary-path errors and measured per-sample cost are also reported.

## 1. Introduction

Active noise control (ANC) suppresses a primary acoustic field by superimposing an anti-phase secondary field and is particularly effective at low frequencies, where passive treatments are often inefficient [1,2,3,4]. The adaptive core of most ANC systems is the filtered-x least-mean-square (FxLMS) algorithm [1], which filters the reference through a secondary-path estimate to preserve the gradient direction in the presence of actuator–sensor dynamics. A centralized multichannel FxLMS controller accounts for all acoustic paths, but its computation and communication grow rapidly with the number of channels [5]. Distributed ANC partitions the system into locally connected nodes and therefore provides a more scalable implementation [6,7].

Diffusion adaptation is a natural framework for distributed ANC: each node first adapts a local estimate and then combines intermediate estimates received from its neighbors [8]. Diffusion FxLMS variants include direct and multitask formulations [9,10,11,12]. In an asymmetric acoustic field, however, neighboring nodes generally have different optimal control filters, so directly averaging unlike filters creates a spatial-smoothing bias. Augmented-diffusion FxLMS (ADFxLMS) avoids this problem by letting each node maintain neighborhood-augmented copies and combining only different estimates of the same physical filter [13]. Bidirectional, block, selective collaboration, and multi-device variants have subsequently reduced its cost or demonstrated its practical feasibility [14,15,16,17]. In parallel, learning-based ANC has advanced rapidly: deep neural networks have been trained as multichannel controllers [18], generative fixed-filter ANC combines a pre-trained network with a Bayesian filter to produce control filters without delay [19], and spatial-frequency selective fixed-filter control extends pre-computed filters to multichannel systems [20]. These methods target fast-varying primary noise and rely on offline training or pre-computed filter banks. The present work instead concerns impulsive measurement disturbances in an adaptive distributed controller.

These developments do not remove the sensitivity of the local mean-square-error update to impulsive disturbances. M-estimation limits the influence of outliers [21], while maximum correntropy criterion (MCC) methods use a Gaussian kernel whose weight decreases for large errors, producing a redescending influence function [22,23,24,25]. MCC-based ANC algorithms have been developed for centralized or single-channel systems [26,27,28,29,30], and recent single-channel work has also reduced the cost of the adaptation, for example, by a filtered-x fast Euclidean direction search built on an augmented-space linear model with low-cost center clustering [31], or has used enhanced long short-term memory controllers with online secondary-path modeling [32]; normalized MCC adaptation has been studied for noisy-input identification [33], and diffusion MCC methods have been studied for distributed estimation and spectrum sensing [34,35,36,37,38,39,40,41]. Adaptive filtering has also been generalized beyond real-valued linear models; for example, widely linear adaptive filters based on Clifford geometric algebra provide a unified framework for multidimensional signals [42]. Robust distributed ANC has also been approached in four other ways. The diffusion algorithm of Zhou et al. [43] (DADFxLMM) uses the augmented weight structure together with a modified-Huber M-estimate whose threshold follows a recursive robust variance estimate [44]. The mixed-gradient algorithm (MGDFxLMS) [45] exchanges compensated gradients to cope with cross-talk and communication delay. Swish-based diffusion [46] uses an activation-function-shaped cost. The self-boosted weight-constrained FxLMS (SB-WCFxLMS) [47] stabilizes decentralized nodes without communication. DADFxLMM is the closest to the present work: it already robustifies augmented diffusion, but with a hard threshold, a fixed rejection constant and a discontinuous influence function. A smooth, scale-equivariant correntropy gate within augmented diffusion, together with a mean-square analysis of the resulting recursion, has not been reported. Robust processing of corrupted or spurious measurements is also a concern in other networked and multisensor systems, for example in radar-guided infrared perception that jointly detects targets and suppresses ghost detections [48], and in the fusion of temporal, Doppler and spatial features of millimeter-wave radar data for direction-insensitive human-activity recognition [49].

This paper therefore retains the admissible augmented-diffusion combination stage and robustifies its local filtered-x adaptation with a scale-adaptive MCC gate. The main contributions are:

- *C1:* A normalized R-ADFxMCC recursion that combines only estimates of the same physical filter and uses a median-absolute-deviation (MAD)-scaled correntropy gate to reject impulsive errors sample by sample. Setting the gate to one recovers normalized ADFxLMS exactly.
- *C2:* A bounded-influence analysis and an approximate mean-convergence model. Under the stated independence and linearization approximations, for any fixed nonnegative block-row-stochastic combination rule, the sufficient mean stability bound depends on the normalized gated regressor statistics rather than on the particular combination coefficients. A symmetry argument establishes zero steady-state mean bias without the independence approximation. Because the correntropy nonlinearity is smooth, a local steady-state excess mean-square error (EMSE) approximation and a mean-square step-size condition also follow; the second-order expansion behind them requires a differentiable nonlinearity and does not apply directly to the hard-threshold M-estimate.
- *C3:* Out-of-sample verification of the mean recursion and of the EMSE expression, and a 30-realization comparison with six centralized, decentralized and distributed baselines, including the M-estimate augmented-diffusion algorithm. Every method’s step size is selected by the same validation protocol on seeds disjoint from the test seeds. Additional tests cover the impulse probability, the kernel-width factor, the MAD window, symmetric α-stable noise, reverberant paths, secondary-path modeling errors and measured per-sample cost.

Section 2 formulates the distributed ANC problem and reviews ADFxLMS. Section 3 develops R-ADFxMCC, Section 4 analyzes robustness and mean convergence, Section 5 reports the simulations, and Section 6 concludes.

Lowercase and uppercase boldface denote vectors and matrices, respectively; ${\left(\cdot \right)}^{T}$ denotes transpose; ∥·∥ is the Euclidean norm; col{·} denotes column stacking; and blkdiag{·} denotes a block-diagonal matrix.

## 2. Problem Formulation

### 2.1. Distributed Multichannel ANC Model

Consider a *K*-node network in which node *k* contains one secondary loudspeaker, one error microphone, and a local processor. The reference signal is x(n), and $x\left(n\right)={\left[x\left(n\right),\ldots ,x\left(n-L+1\right)\right]}^{T}$. The local control filter $w_{k}\left(n\right)\in R^{L}$ produces

(1) $$u_{k}\left(n\right)=w_{k}^{T}\left(n\right)x\left(n\right).$$

Let $s_{ki}\in R^{J}$ be the secondary path from loudspeaker *i* to error microphone *k*, and let $u_{i}\left(n\right)={\left[u_{i}\left(n\right),\ldots ,u_{i}\left(n-J+1\right)\right]}^{T}$. The control sound and measured error are

(2) $$\begin{array}{cc} y_{k}\left(n\right) & =\sum_{i=1}^{K}s_{ki}^{T}u_{i}\left(n\right),\\ e_{k}\left(n\right) & =d_{k}\left(n\right)+y_{k}\left(n\right)+\nu_{k}\left(n\right), \end{array}$$

where $d_{k}\left(n\right)$ is the primary disturbance and $\nu_{k}\left(n\right)$ is measurement noise. Although the physical acoustic sum includes every loudspeaker, a distributed update exchanges information only within the neighborhood $N_{k}$, which includes node *k*. The topology should therefore cover the dominant coupling paths; weak unmodeled far-field interactions are treated as residual disturbances [13,50].

### 2.2. Impulsive-Noise Models

Two standard impulsive models are considered [4,26]. In Bernoulli–Gaussian (BG) noise, $\nu_{k}\left(n\right)=\eta_{k}\left(n\right)+b_{k}\left(n\right)\zeta_{k}\left(n\right)$, where $\eta_{k}\left(n\right)∼N\left(0,\sigma_{\eta }^{2}\right)$, $b_{k}\left(n\right)∼\mathrm{Bernoulli}\left(p_{r}\right)$, and $\zeta_{k}\left(n\right)∼N\left(0,\sigma_{\zeta }^{2}\right)$ are mutually independent with $\sigma_{\zeta }^{2}\gg \sigma_{\eta }^{2}$. A symmetric α-stable (SαS) disturbance has characteristic exponent α∈(0,2]; smaller α indicates a heavier tail, and its variance is infinite when α<2. Both models are used in the simulations (Section 5.3, Section 5.4 and Section 5.5). Under either model, a squared-error update can be dominated by a small number of extreme samples.

### 2.3. Augmented-Diffusion FxLMS

At node *k*, define the augmented state $\xi_{k}\left(n\right)=\mathrm{col}{\left\{{\tilde{w}}_{l}^{\;k}\left(n\right)\right\}}_{l\in N_{k}}$, where ${\tilde{w}}_{l}^{\;k}$ is node *k*’s copy of the physical control filter at node *l*. The corresponding filtered-reference vector is ${\hat{r}}_{F,k}\left(n\right)=\mathrm{col}{\left\{{\hat{x}}_{F,kl}\left(n\right)\right\}}_{l\in N_{k}}$, where ${\hat{x}}_{F,kl}\left(n\right)={\hat{s}}_{kl}^{T}x_{J}\left(n\right)$, ${\hat{s}}_{kl}$ is the estimate of $s_{kl}$, $x_{J}\left(n\right)={\left[x\left(n\right),\ldots ,x\left(n-J+1\right)\right]}^{T}$, and ${\hat{x}}_{F,kl}\left(n\right)={\left[{\hat{x}}_{F,kl}\left(n\right),\ldots ,{\hat{x}}_{F,kl}\left(n-L+1\right)\right]}^{T}$.

A normalized adapt-then-combine ADFxLMS recursion is

(3) $$\varphi_{k}\left(n+1\right)=\xi_{k}\left(n\right)-\lambda_{k}\frac{{\hat{r}}_{F,k}\left(n\right)e_{k}\left(n\right)}{∥{\hat{r}}_{F,k}{\left(n\right)∥}^{2}+\epsilon_{r}},$$

(4) $$\begin{array}{cc} \xi_{k}\left(n+1\right) & =\sum_{p\in N_{k}}C^{kp}\varphi_{p}\left(n+1\right),\\ w_{k}\left(n+1\right) & =G_{k}^{k}\xi_{k}\left(n+1\right), \end{array}$$

where $G_{k}^{k}$ extracts node *k*’s own filter. With fixed ordered neighborhood lists, where $N_{k}\left(m\right)$ denotes the *m*th entry of the list of node *k*, the block coefficient in $C^{kp}$ is nonzero only when its target and source blocks represent the same physical filter. The scalar combination coefficients satisfy

(5) $$\begin{array}{c} c_{m,q}^{kp}\geq 0,\;c_{m,q}^{kp}=0\;\mathrm{if}\;N_{k}\left(m\right)\neq N_{p}\left(q\right),\\ \sum_{p\in N_{k}}\;\sum_{\begin{array}{c} q:\;N_{p}\left(q\right)=N_{k}\left(m\right) \end{array}}c_{m,q}^{kp}=1. \end{array}$$

Thus the combination stage is block-row-stochastic and preserves the physical meaning of every augmented slot. The remaining weakness is the unbounded error factor in Equation (3).

## 3. Proposed R-ADFxMCC

### 3.1. Correntropy-Gated Robust Adaptation

For a positive kernel width $\sigma_{k}\left(n\right)$, consider the MCC loss

(6) $$J_{k}^{\mathrm{MCC}}=\sigma_{k}^{2}\left(n\right)\left(1-E\left[\exp\left(-\frac{e_{k}^{2}\left(n\right)}{2\sigma_{k}^{2}\left(n\right)}\right)\right]\right).$$

Define the correntropy gate $\varrho_{\sigma_{k}\left(n\right)}\left(e\right)=\exp\left[-e^{2}/\left(2\sigma_{k}^{2}\left(n\right)\right)\right]$. Under the usual filtered-x approximation and a quasi-static kernel width during one update, the proposed robust adaptation is

(7) $$\varphi_{k}\left(n+1\right)=\xi_{k}\left(n\right)-\lambda_{k}\frac{\varrho_{\sigma_{k}\left(n\right)}\left(e_{k}\left(n\right)\right){\hat{r}}_{F,k}\left(n\right)e_{k}\left(n\right)}{∥{\hat{r}}_{F,k}{\left(n\right)∥}^{2}+\epsilon_{r}}.$$

Small errors have ϱ≈1 and therefore receive an ADFxLMS-like update, whereas a large impulsive error is exponentially suppressed. The combination and extraction stages remain exactly those of Equations (4) and (5); no learned parameters or data-dependent combination weights are introduced.

### 3.2. Adaptive Kernel Width

A fixed $\sigma_{k}$ is difficult to choose when residual levels differ among nodes or change during adaptation. R-ADFxMCC estimates a robust local scale from the most recent $N_{w}$ errors:

(8) $$\begin{array}{cc} {\hat{\varsigma }}_{k}\left(n\right) & =1.4826\;{\mathrm{median}}_{i\in W_{n}}\left|e_{k}\left(i\right)-{\mathrm{median}}_{j\in W_{n}}e_{k}\left(j\right)\right|,\\ \sigma_{k}\left(n\right) & =c_{\sigma }\max\left({\hat{\varsigma }}_{k}\left(n\right),\varsigma_{\min}\right), \end{array}$$

where $W_{n}$ is the available window of at most $N_{w}$ samples, $c_{\sigma }>0$ sets the nominal outlier boundary, and $\varsigma_{\min}>0$ prevents a zero kernel width. The factor 1.4826 makes the median absolute deviation consistent with the standard deviation for Gaussian data. In the implementation, the MCC gate is set to one during the first eight samples, while the scale window is initialized.

### 3.3. Gradient Interpretation and Limiting Cases

For one sample, let $\ell_{k}\left(n\right)=\sigma_{k}^{2}\left(n\right)\left[1-\exp\left(-e_{k}^{2}\left(n\right)/\left(2\sigma_{k}^{2}\left(n\right)\right)\right)\right]$. Treating $\sigma_{k}\left(n\right)$ as quasi-static over the current update and using the filtered-x approximation gives

(9) $$\nabla_{\xi_{k}}\ell_{k}\left(n\right)≃\varrho_{\sigma_{k}\left(n\right)}\left(e_{k}\left(n\right)\right)e_{k}\left(n\right){\hat{r}}_{F,k}\left(n\right).$$

Equation (7) is therefore a normalized stochastic-gradient step on the local MCC loss. If $\Delta \varphi_{k}^{\mathrm{MSE}}$ denotes the normalized ADFxLMS (mean-square error, MSE) increment computed from the same error and regressor, then

(10) $$\frac{∥\Delta \varphi_{k}^{\mathrm{MCC}}∥}{∥\Delta \varphi_{k}^{\mathrm{MSE}}∥}=\varrho_{\sigma_{k}\left(n\right)}\left(e_{k}\left(n\right)\right)\in \left(0,1\right].$$

Thus robustness is introduced by a scalar contraction of the local update, not by changing the augmented coordinates or the combination rule. This separation is useful in implementation because an existing normalized ADFxLMS system needs only the scale estimator and gate inserted before its local gradient.

Several familiar algorithms are recovered as limiting cases. As $\sigma_{k}\to \infty$, the gate approaches one and Equation (7) becomes normalized ADFxLMS. With a singleton neighborhood, the augmented state contains only the local filter and the recursion becomes normalized filtered-x MCC (FxMCC). With K=1, it further reduces to the conventional single-channel normalized filtered-x MCC update. Finally, when the residual stays within a small fraction of the kernel width, a second-order expansion gives $\varrho \left(e\right)=1-e^{2}/\left(2\sigma_{k}^{2}\right)+O\left(e^{4}/\sigma_{k}^{4}\right)$, explaining why the proposed method closely tracks the MSE update in nonimpulsive conditions.

*Relation to the M-estimate update.* DADFxLMM [43] replaces $\varrho_{\sigma_{k}}\left(e\right)e$ by the modified-Huber function ψ(e)=e for $\left|e\right|\leq \tau_{k}$ and ψ(e)=0 otherwise, with $\tau_{k}=2.576\;{\hat{\sigma }}_{e,k}$ and a recursive median-based variance estimate [44]. Both updates are redescending and share the augmented-diffusion structure. The differences are that the correntropy gate is smooth (so the EMSE analysis of Section 4.5 applies), and that its only parameters are $c_{\sigma }$, a multiplicative factor on a MAD estimate, and the window length $N_{w}$, with no rejection constant or forgetting factor. Section 5.3 compares the two directly.

### 3.4. Algorithm Summary

Table 1 lists one iteration of R-ADFxMCC at node *k*. Step 0 initializes the augmented state and fixes the step size $\lambda_{k}$, the kernel-width factor $c_{\sigma }$, the MAD window length $N_{w}$ and the regularization $\epsilon_{r}$. At each sample, the node measures its error and updates the kernel width $\sigma_{k}\left(n\right)$ from the most recent $N_{w}$ errors by Equation (8) (Step 1); during the first eight samples, the gate is set to one. It then forms the filtered-reference vector and performs the correntropy-gated normalized adaptation of Equation (7) (Step 2). The intermediate augmented estimates are exchanged with the neighbors and combined slot by slot with the fixed admissible coefficients of Equations (4) and (5) (Step 3), and the node’s own control filter is extracted to generate the next control output (Step 4). Only Steps 1 and 2 differ from ADFxLMS; Steps 3 and 4 are unchanged, so the communication pattern and payload are the same as in ADFxLMS (Section 3.5).

### 3.5. Implementation Cost and Communication

Table 2 separates the adaptive state, local arithmetic, and communication payload. Here $\delta_{k}=\left|N_{k}\right|$, and MDFxLMS is the conventional diffusion FxLMS described in Section 5.1. The order notation excludes secondary-path filtering, error sensing, and acoustic output generation, which are common to the compared distributed algorithms.

Relative to normalized ADFxLMS, R-ADFxMCC adds one Gaussian kernel evaluation, a ring buffer of $N_{w}$ residuals, and the robust scale update. A direct implementation that sorts the window costs $O(N_{w}\log N_{w})$ comparisons; selection-based order statistics reduce the expected cost to $O(N_{w})$ without changing the recursion. The two medians in Equation (8) can reuse the same scratch storage. The scale need not be recomputed at audio rate: updating it once every *P* samples and holding $\sigma_{k}$ between updates reduces the cost of the scale update (the two medians) by approximately *P* while preserving the sample-wise gate evaluation.

To replace the order-of-magnitude argument with a measurement, the local operations of one node-sample were implemented in C (quickselect medians on a scratch copy of the window, the library exp, and the uniform combination of $\delta_{k}$ slots with $\delta_{k}$ contributors each). They were timed over $2\times 10^{6}$ iterations on one core of an Intel Xeon processor (2.10 GHz), compiled with gcc -O2. A node-sample took 835 ns for ADFxLMS, 1733 ns for DADFxLMM and 2790 ns for R-ADFxMCC. The correntropy gate with two medians therefore adds 1955 ns, and the whole local update uses 1.40% of the 200 μs sample period at 5 kHz. On an embedded processor, the absolute times scale with the clock rate and memory system, but the ratio of about 3.3 between the local updates of R-ADFxMCC and ADFxLMS indicates the relative cost. Updating $\sigma_{k}$ every *P* samples reduces the median part of the overhead to about 1/P of this value; the exponential is still evaluated at every sample. The timed operations are those of the local update in Table 2: the regressor energy, the normalized (gated) update of the $\delta_{k}L$ augmented weights, the combination of the $\delta_{k}$ slots and, for the robust methods, the scale estimate and the gate. They exclude the filtered-reference computation, the control output filter, sensor and actuator I/O, and the transmission of the intermediate vectors. To relate the local update to the complete per-node processing, the benchmark was repeated with the reference-buffer update, the control output $u_{k}\left(n\right)$ (*L* multiply–accumulate operations), the $\delta_{k}$ filtered references ($\delta_{k}J$ multiply–accumulate operations, J=36) and the shift of the filtered-reference regressors added. In this run, the local update took 814, 1726 and 2838 ns, and the complete per-node processing took 1000, 1881 and 2968 ns for ADFxLMS, DADFxLMM and R-ADFxMCC, i.e., 0.50%, 0.94% and 1.48% of the sample period. These times still exclude I/O and communication, whose cost depends on the hardware and network and is the same for the three methods because the transmitted payload is unchanged.

Most importantly, R-ADFxMCC does not increase network traffic. Each node transmits the same intermediate augmented vector as ADFxLMS, with no side channel for residuals, kernel widths, or noise statistics. The method is consequently attractive when communication, rather than arithmetic, is the limiting resource. The extra memory is only $N_{w}$ scalars per node and is independent of the control filter length and neighborhood degree.

## 4. Theoretical Analysis

### 4.1. Bounded Influence

The scalar influence function of the instantaneous MCC loss and its limiting properties are

(11) $$\begin{array}{cc} \psi_{\sigma_{k}}\left(e\right) & =e\exp[-e^{2}/\left(2\sigma_{k}^{2}\right)],\\ \max_{e}\left|\psi_{\sigma_{k}}\left(e\right)\right| & =\sigma_{k}e^{-1/2}\approx 0.6065\sigma_{k},\\ \lim_{|e|\to \infty }\psi_{\sigma_{k}}\left(e\right) & =0. \end{array}$$

Since $∥r∥/{(∥r∥}^{2}+\epsilon_{r})\leq 1/\left(2\sqrt{\epsilon_{r}}\right)$ for any vector r, the increment in Equation (7) obeys

(12) $$∥\varphi_{k}\left(n+1\right)-\xi_{k}\left(n\right)∥\leq \frac{0.6065\lambda_{k}\sigma_{k}\left(n\right)}{2\sqrt{\epsilon_{r}}}.$$

Thus every individual update is bounded whenever $\sigma_{k}\left(n\right)$ is finite, and the contribution of an extreme error decreases to zero.

The attenuation has a direct engineering interpretation. For an error of magnitude $a\sigma_{k}$, the robust-to-MSE update ratio in Equation (10) is $\exp(-a^{2}/2)$. It equals 0.6065, 0.1353, 0.0111, and $3.35\times 10^{-4}$ for a=1, 2, 3, and 4, respectively. A four-kernel-width sample therefore contributes less than 0.04% of the corresponding normalized MSE update. Squaring Equation (12) also gives the deterministic update-energy bound

$$∥\varphi_{k}\left(n+1\right)-\xi_{k}{\left(n\right)∥}^{2}\leq 0.0920\;\frac{\lambda_{k}^{2}\sigma_{k}^{2}\left(n\right)}{\epsilon_{r}},$$

which is valid sample by sample and does not require a finite variance for the instantaneous contamination.

### 4.2. Scale Robustness Under Contaminated Windows

The gate is useful only if an outlier cannot enlarge its own kernel width enough to cancel the rejection. The MAD in Equation (8) has a 50% breakdown point: fewer than half of the window samples can move arbitrarily far without forcing the estimate to infinity. For independent Bernoulli–Gaussian impulses with $p_{r}=0.10$ and $N_{w}=64$, the expected number of contaminated samples is 6.4, well below the 32 samples required to reach this breakdown fraction. Moreover, the estimate is local, so impulses confined to a few microphones do not change the widths at uncontaminated nodes.

This argument also identifies a limitation. A long burst occupying at least half of a node’s window can inflate ${\hat{\varsigma }}_{k}$, broaden the kernel, and temporarily weaken rejection. Reducing $N_{w}$ shortens recovery after such a burst but makes the scale noisier; increasing $N_{w}$ does the opposite. The sensitivity experiment in Section 5.4 therefore evaluates a threefold range of window lengths. The floor $\varsigma_{\min}$ addresses the complementary clean-start problem: without it, an all-zero initial window would produce an undefined exponent.

### 4.3. Mean-Convergence Condition

The analysis compares the adaptive weights with an ideal solution. Let $w_{l}^{o}$ denote the ideal value of the physical control filter at node *l*, i.e., the value that minimizes the network mean-square error, and let $y_{k}^{o}\left(n\right)$ be the control signal at microphone *k* when every node uses its ideal filter. The ideal augmented solution of node *k* stacks the ideal filters of its neighborhood in the same order as $\xi_{k}\left(n\right)$, $\xi_{k}^{o}=\mathrm{col}{\left\{w_{l}^{o}\right\}}_{l\in N_{k}}$. The network augmented state is $\Gamma^{A}\left(n\right)=\mathrm{col}{\left\{\xi_{k}\left(n\right)\right\}}_{k=1}^{K}$, its ideal value is $\Gamma^{A,o}=\mathrm{col}{\left\{\xi_{k}^{o}\right\}}_{k=1}^{K}$, and ${\tilde{\Gamma }}^{A}\left(n\right)=\Gamma^{A}\left(n\right)-\Gamma^{A,o}$. The residual error at the ideal solution is

$$v_{k}\left(n\right)=d_{k}\left(n\right)+y_{k}^{o}\left(n\right)+\nu_{k}\left(n\right),$$

which comprises the measurement noise $\nu_{k}\left(n\right)$ and any part of the primary disturbance that the ideal filters cannot cancel. Under (A1) below, the ideal solution cancels the primary disturbance and $v_{k}\left(n\right)=\nu_{k}\left(n\right)$.

The analysis uses the following assumptions. The results that depend on each assumption are stated after the list:

(A1) *Linear error model.* The secondary-path estimates are exact, the neighborhoods cover the acoustic coupling, and an ideal augmented solution cancels the primary disturbance, so that the error of every node is linear in the current and recent stacked weight errors, $e_{k}\left(n\right)=\sum_{j=0}^{D}h_{k,j}^{T}\left(n\right){\tilde{\Gamma }}^{A}\left(n-j\right)+v_{k}\left(n\right)$, where $h_{k,j}\left(n\right)$ depends only on the reference and *D* covers the secondary-path delay.
(A2) *Noise.* The residual disturbance $v_{k}\left(n\right)$ at the ideal solution is independent of the reference and white in time.
(A3) *Separation.* Within one update, the filtered regressor, the gate and the weight error are treated as mutually independent, and $\sigma_{k}\left(n\right)$ is treated as given.

The bounded-influence results above need none of these assumptions. The zero-bias result in Section 4.4 needs (A1), (A2) and a symmetry condition, but not (A3). Only the mean recursion below and the EMSE expression in Section 4.5 use (A3). Under (A1)–(A3), and with fixed combination coefficients satisfying Equation (5), step sizes $\lambda_{k}$ that are constant during a run, and filtered regressors with finite second moments [8,35,36], the forcing term has zero mean at the ideal augmented solution:

$$E\left[\varrho_{\sigma_{k}\left(n\right)}\left(e_{k}\left(n\right)\right)\frac{{\hat{r}}_{F,k}\left(n\right)v_{k}\left(n\right)}{∥{\hat{r}}_{F,k}{\left(n\right)∥}^{2}+\epsilon_{r}}\right]=0.$$

Let ${\tilde{\xi }}_{k}\left(n\right)=\xi_{k}\left(n\right)-\xi_{k}^{o}$, ${\tilde{\varphi }}_{k}\left(n\right)=\varphi_{k}\left(n\right)-\xi_{k}^{o}$ and define ${\bar{P}}_{k}\left(n\right)={\hat{r}}_{F,k}\left(n\right){\hat{r}}_{F,k}^{T}\left(n\right)/(∥{\hat{r}}_{F,k}{\left(n\right)∥}^{2}$$+\epsilon_{r})$.

Under the usual filtered-x linearization, substitution in Equation (7) gives

(13) $$\begin{array}{cc} {\tilde{\varphi }}_{k}\left(n+1\right)= & \left[I-\lambda_{k}\varrho_{\sigma_{k}\left(n\right)}\left(e_{k}\left(n\right)\right){\bar{P}}_{k}\left(n\right)\right]{\tilde{\xi }}_{k}\left(n\right)\\ \; & -\lambda_{k}\varrho_{\sigma_{k}\left(n\right)}\left(e_{k}\left(n\right)\right)\frac{{\hat{r}}_{F,k}\left(n\right)v_{k}\left(n\right)}{∥{\hat{r}}_{F,k}{\left(n\right)∥}^{2}+\epsilon_{r}}. \end{array}$$

Define the normalized gated correlation matrix

(14) $${\bar{R}}_{\varrho ,k}\left(n\right)=E\left[\varrho_{\sigma_{k}\left(n\right)}\left(e_{k}\left(n\right)\right){\bar{P}}_{k}\left(n\right)\right].$$

Taking expectations yields the approximate local recursion

(15) $$E\left[{\tilde{\varphi }}_{k}\left(n+1\right)\right]≃\left[I-\lambda_{k}{\bar{R}}_{\varrho ,k}\left(n\right)\right]E\left[{\tilde{\xi }}_{k}\left(n\right)\right].$$

Stacking all augmented errors and applying the fixed combination matrix $C^{A}$ gives

(16) $$\begin{array}{cc} E[{\tilde{\Gamma }}^{A}\left(n+1\right)]≃ & C^{A}\;\mathrm{blkdiag}{\left\{I-\lambda_{k}{\bar{R}}_{\varrho ,k}\left(n\right)\right\}}_{k=1}^{K}\\ \; & \times E[{\tilde{\Gamma }}^{A}\left(n\right)]. \end{array}$$

Because Equation (5) makes every slot a convex combination of estimates of the same physical filter, $∥C^{A}{∥}_{b,\infty }=1$ in the compatible augmented block-maximum norm. The combination is therefore nonexpansive. Under sufficient excitation, a node-wise sufficient condition for stability of the approximate mean recursion in Equation (16), which holds under (A1)–(A3) and the filtered-x linearization, is

(17) $$0<\lambda_{k}<\frac{2}{\theta_{\max}\left({\bar{R}}_{\varrho ,k}\left(n\right)\right)}\;\mathrm{for}\;\mathrm{all}\;k,n.$$

where $\theta_{\max}\left(\cdot \right)$ denotes the largest eigenvalue. Moreover, 0<ϱ≤1 and $\epsilon_{r}>0$ imply $\theta_{\max}\left({\bar{R}}_{\varrho ,k}\right)<1$ for the normalized update. Hence $0<\lambda_{k}<2$ is a conservative statistics-independent sufficient condition within the same approximations; it is not an exact stability guarantee for the nonlinear, delayed closed loop. The particular admissible combination coefficients do not enter this bound, although they still affect convergence speed and disagreement attenuation. As $\sigma_{k}\to \infty$, ϱ→1 and the recursion reduces to normalized ADFxLMS. Equation (17) concerns the mean only and does not account for the delay between the control output and the error in the filtered-x loop, which in practice lowers the usable step size (Section 5.3).

### 4.4. Steady-State Mean Bias

The zero-mean forcing term above factorizes the expectation and therefore relies on (A3). Because the gate is computed from the same residual that enters the update, the factorization is not exact. The following symmetry argument avoids it.

**Proposition** **1.** *Assume (A1) and (A2), and suppose that the joint process formed by the recent weight errors ${\left\{{\tilde{\Gamma }}^{A}\left(n-j\right)\right\}}_{j=0}^{D}$, the $N_{w}$-sample error windows, and the reference window admits a unique stationary distribution with a finite first moment: (i) If the residual sequence is sign-symmetric, i.e., $\left\{-v_{k}\left(n\right)\right\}$ has the same joint law as $\left\{v_{k}\left(n\right)\right\}$, then $E[{\tilde{\Gamma }}^{A}]=0$ and $E[e_{k}-v_{k}]=0$ in a steady state. (ii) If instead the reference is sign-symmetric, then $E[{\tilde{\Gamma }}^{A}]=0$ for every residual distribution that is independent of the reference.*

**Proof.**  Let T be one iteration of R-ADFxMCC acting on this state and driven by {x,v}. Under (A1), the error is linear in the weight-error history and *v*. Replacing the weight-error history and *v* with their negatives changes the sign of $e_{k}$ and of every window. The window median changes the sign, and the MAD is unchanged, so $\sigma_{k}\left(n\right)$ and the even gate are unchanged. The increment in Equation (7) is odd in $e_{k}$, and the combination is linear. The negated state is therefore a solution of T driven by {x,−v}, which under (i) has the same law as {x,v}. Uniqueness of the stationary law implies that it is invariant under negation, so the weight error and the a priori error $e_{k}-v_{k}$ have zero mean. For (ii), negating the weight-error history and the reference leaves every $e_{k}$ and $\sigma_{k}$ unchanged and negates every increment, and the same reasoning applies. □

Proposition 1 needs no independence between the gate, the regressor and the weight error, so it does not rely on the factorization used above. It also shows when a bias can occur: when neither the residual nor the reference is sign-symmetric, or when the residual contains a component that is correlated with the reference, such as an uncancellable part of $d_{k}$ caused by noncausal or unmodeled paths. In case (ii) alone, the weight error is unbiased but the a priori error may acquire a small offset proportional to $E[\psi_{\sigma_{k}}\left(v_{k}\right)]$. A normalized LMS update has no such offset for zero-mean noise, because its error nonlinearity is linear. Section 5.2 confirms both cases, and the case in which neither symmetry holds, numerically.

### 4.5. Mean-Square Behavior and Steady-State EMSE

Mean convergence does not describe the fluctuation of the weights under impulsive contamination. Following the energy-conservation analysis of adaptive filters with error nonlinearities [51], write $f\left(e\right)=\psi_{\sigma_{k}}\left(e\right)=e\;\varrho_{\sigma_{k}}\left(e\right)$ and define the local a priori error $e_{a,k}\left(n\right)={\hat{r}}_{F,k}^{T}\left(n\right){\tilde{\xi }}_{k}\left(n\right)$. This definition treats node *k*’s copies of its neighbors’ filters as equal to the filters actually in use; i.e., it neglects the disagreement between copies. In the limit $\epsilon_{r}\to 0$, Equation (7) satisfies the energy relation

(18) $$∥{\tilde{\varphi }}_{k}{\left(n+1\right)∥}^{2}={∥{\tilde{\xi }}_{k}\left(n\right)∥}^{2}-\frac{2\lambda_{k}e_{a,k}\left(n\right)f\left(e_{k}\left(n\right)\right)}{∥{\hat{r}}_{F,k}{\left(n\right)∥}^{2}}+\frac{\lambda_{k}^{2}f^{2}\left(e_{k}\left(n\right)\right)}{∥{\hat{r}}_{F,k}{\left(n\right)∥}^{2}}.$$

In steady state, and neglecting the effect of the combination stage on the local energy, the expected energies on both sides are equal. Under (A3), the regressor energy separates from the errors, which gives $2E\left[e_{a,k}f\left(e_{k}\right)\right]=\lambda_{k}E\left[f^{2}\left(e_{k}\right)\right]$. Expanding $f(e_{a,k}+v_{k})$ to the second order around $v_{k}$, with $e_{a,k}$ small, zero-mean and independent of $v_{k}$, yields the local steady-state excess mean-square error (EMSE) approximation

(19) $$E_{k}=E\left[e_{a,k}^{2}\right]≃\frac{\lambda_{k}A_{k}}{2B_{k}-\lambda_{k}C_{k}},$$

where $A_{k}=E\left[f^{2}\left(v_{k}\right)\right]$, $B_{k}=E\left[f^{\prime }\left(v_{k}\right)\right]$ and $C_{k}=E\left[f^{\prime 2}\left(v_{k}\right)+f\left(v_{k}\right)f^{\prime \prime }\left(v_{k}\right)\right]$, and $\sigma_{k}$ is fixed at its steady-state value, $c_{\sigma }$ times the MAD scale of $v_{k}$. For the Gaussian kernel, $f^{\prime }\left(v\right)=\left(1-v^{2}/\sigma_{k}^{2}\right)\varrho$ and $f^{\prime 2}+ff^{\prime \prime }=\left(1-5v^{2}/\sigma_{k}^{2}+2v^{4}/\sigma_{k}^{4}\right)\varrho^{2}$. The expression is meaningful only for $B_{k}>0$, i.e., when the gated update is on average a descent step for the a priori error, and a positive EMSE then requires $2B_{k}-\lambda_{k}C_{k}>0$. If $C_{k}>0$, this gives the mean-square condition $\lambda_{k}<2B_{k}/C_{k}$, and together with the statistics-independent condition $0<\lambda_{k}<2$ that follows from Equation (17), the admissible range within the approximation is $0<\lambda_{k}<\min\left\{2,2B_{k}/C_{k}\right\}$. If $C_{k}\leq 0$, the denominator is positive for every $\lambda_{k}>0$, the mean-square condition adds no restriction, and only Equation (17) applies. For a zero-mean Gaussian residual with variance $\sigma_{v,k}^{2}$ and $r_{k}=\sigma_{v,k}^{2}/\sigma_{k}^{2}$, direct integration gives $B_{k}={\left(1+r_{k}\right)}^{-3/2}>0$ and $C_{k}=\left(1-r_{k}\right){\left(1+2r_{k}\right)}^{-5/2}$, so $C_{k}>0$ exactly when the kernel width exceeds the residual standard deviation. For a Gaussian mixture such as the BG model, $B_{k}$ and $C_{k}$ are the probability-weighted sums of the component values; hence $B_{k}>0$ always, and an impulsive component with $r_{k}\gg 1$ contributes only a negative term of order $r_{k}^{-3/2}$ to $C_{k}$. For f(e)=e (normalized ADFxLMS), $A_{k}=\sigma_{v,k}^{2}=E\left[v_{k}^{2}\right]$ and $B_{k}=C_{k}=1$, and Equation (19) reduces to the classical $\lambda_{k}\sigma_{v,k}^{2}/\left(2-\lambda_{k}\right)$.

Equation (19) makes the robustness mechanism quantitative. For the MSE update, $A_{k}$ is the total noise variance, which impulses dominate: with $p_{r}=0.06$ and $\sigma_{\zeta }^{2}/\sigma_{\eta }^{2}=2\times 10^{4}$, it is $1+0.06\times 2\times 10^{4}=1201$ times the background variance. For the correntropy update, $f^{2}\left(v\right)=v^{2}\exp\left(-v^{2}/\sigma_{k}^{2}\right)$ vanishes for large |v|, so $A_{k}$, $B_{k}$ and $C_{k}$ are set almost entirely by the background noise. Because $0<B_{k}<1$, the gate also lowers the effective step size by the factor $B_{k}$. This is consistent with the larger stable step-size range observed in Section 5.3, although the delayed filtered-x loop, which Equation (19) does not model, determines the actual stability limit. Equation (19) is a local approximation: it treats node *k* in isolation and neglects the combination stage and the disagreement between copies. It is not a proven upper bound on the network EMSE. Because the combination averages the fluctuations of several copies of each physical filter, the network EMSE is expected to be smaller than $E_{k}$. In the network of Section 5.2, the expression exceeded the simulated network EMSE by 1.2–5.7 dB in all 18 cases, with offsets of the same order for the MSE and correntropy updates. This is an empirical observation for that network rather than a general guarantee; the expression is used to describe the dependence of the EMSE on $\lambda_{k}$, $c_{\sigma }$ and the impulse statistics.

### 4.6. Time-Varying Scale and Parameter Guidance

**Figure 1:**
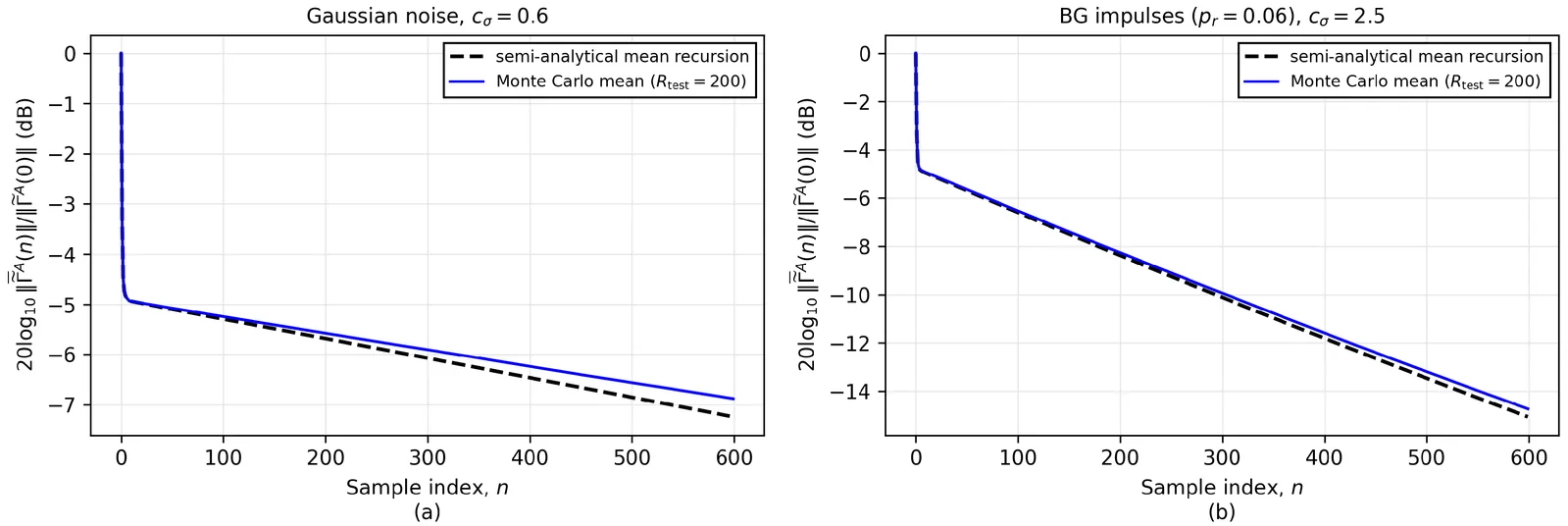
Mean-convergence verification of the correntropy-gated augmented-diffusion recursion: (**a**) Gaussian measurement noise, $c_{\sigma }=0.6$; (**b**) Bernoulli–Gaussian impulses, $c_{\sigma }=2.5$.

The analysis above conditions on the kernel width over one update. This is a standard separation approximation: the filter state changes by one normalized stochastic-gradient step, whereas $\sigma_{k}\left(n\right)$ is a statistic of an $N_{w}$-sample window. The bounded-increment result remains exact for every realized finite $\sigma_{k}\left(n\right)$, but the mean recursion is approximate because the window, gate, regressor, and weight error are not strictly independent. This dependence is one reason for verifying Equation (16) against an independently generated Monte Carlo mean rather than presenting the recursion as an equality. The dependence can also be quantified. When one sample enters the window and the oldest one leaves, the new window median lies between the order statistics $x_{\left(N_{w}/2-1\right)}$ and $x_{\left(N_{w}/2+2\right)}$ of the old window (for even $N_{w}$), so it moves by at most their spread $g_{e}=x_{\left(N_{w}/2+2\right)}-x_{\left(N_{w}/2-1\right)}$. Because the absolute deviations are measured from this median, all retained deviations change as well, but each by no more than the median shift, so their median also moves by at most this amount. Replacing the deviation in the departing sample with that in the new one moves the median of the deviations by at most $g_{d}$, the corresponding spread of the absolute deviations of the old window about its median. Hence $|{\hat{\varsigma }}_{k}\left(n\right)-{\hat{\varsigma }}_{k}\left(n-1\right)|\leq 1.4826\;\left(g_{e}+g_{d}\right)$, a bound that does not depend on the magnitude of the incoming residual, so a single residual has only a bounded effect on its own kernel width, however large it is. In the network of Section 5.2, $\sigma_{k}\left(n\right)$ changed from one sample to the next by a median of 1.9% (99th percentile 12.2%), and by 1.9% (99th percentile 12.8%) on samples that carried an impulse. The sample correlation between the gate value and the regressor energy was 0.003. These small-scale changes support the quasi-static approximation, and the low correlation, together with the agreement in Figure 1 and Table 3, is empirical support for the separation approximation (A3) in the regime considered. It is not evidence of independence: uncorrelated variables can still be dependent, and the gate also depends on the weight error through $e_{k}\left(n\right)$. Both approximations become questionable when bursts occupy a large part of the window (Section 4.2) or when $N_{w}$ is so short that one sample materially changes the median.

The two scale parameters have distinct roles. The factor $c_{\sigma }$ specifies where the transition from MSE-like adaptation to outlier rejection occurs. A very small value rejects ordinary residual samples and slows convergence; a very large value admits more impulsive energy. The window $N_{w}$ controls statistical variability and response time but not the nominal rejection boundary. These roles allow a simple design procedure: first select $N_{w}$ long enough to estimate the background scale but shorter than the expected nonstationarity interval, then choose $c_{\sigma }$ so that typical residuals lie inside the near-linear part of the influence function, and finally choose $\lambda_{k}$ within Equation (17) to meet the desired convergence-speed margin [52], keeping in mind that this condition rests on (A1)–(A3) and does not model the filtered-x delay, so the usable step is found by validation (Section 5.1).

Because the combination stage is nonexpansive, a conservative deployment can use the same $\lambda_{k}$ at every node. Node-dependent steps remain admissible, but they are not necessary for robustness and were not used in the experiments. Likewise, $c_{\sigma }$ and $N_{w}$ may be shared across nodes even when their absolute residual powers differ, since the MAD normalizes the kernel width locally. This scale equivariance is important in asymmetric acoustic fields where a single fixed absolute kernel width would favor either the high-energy or low-energy microphones.

## 5. Simulation Results

### 5.1. Setup

The simulated system has K=10 nodes placed on a ring of radius 1.5 m. Each node comprises a loudspeaker and an error microphone separated by 0.15 m. The communication graph is the ring augmented by links 0–5 and 4–8. The sampling frequency is 5 kHz, the run length is N=12000, the control filter length is L=64, the secondary-path length is J=36, and the primary-path length is $J_{p}=64$. The primary source is at (0,2) m. Except in Section 5.5, the primary and secondary paths are single-tap delayed free-field responses with gain 1/(4πr), where *r* is the source–microphone distance, and the secondary-path estimates are exact. Secondary paths whose delay exceeds J=36 samples (loudspeaker–microphone distances above about 2.4 m) are set to zero, so the weakest far-field couplings are omitted. The measurement noise $\nu_{k}\left(n\right)$ consists of white Gaussian background noise at 30 dB below each node’s primary power plus, unless stated otherwise, BG impulses with $p_{r}=0.06$ and $\sigma_{\zeta }^{2}/\sigma_{\eta }^{2}=2\times 10^{4}$ at nodes {0,1,2}.

*Compared algorithms.* Seven algorithms are compared: centralized normalized multichannel FxLMS (CFxLMS), in which the update of each actuator is normalized by the total filtered-reference energy of that actuator; MDFxLMS, the conventional diffusion FxLMS in which each node adapts its own filter with its own filtered reference and then averages the filters of its neighbors [9,10]; decentralized SB-WCFxLMS [47] (penalty γ; self-boosting period 1000 samples, i.e., 0.2 s instead of the 1 s in [47], because a run lasts 2.4 s); MGDFxLMS [45], implemented with ideal compensation (each node receives the exact cross-path filtered-reference gradients of its neighbors) and, unlike the original fixed-step form, normalized by the total filtered-reference energy so that one grid serves all spectra; normalized ADFxLMS; DADFxLMM [43], implemented in the same normalized augmented-diffusion form as ADFxLMS, with the modified-Huber function, $\tau_{k}=2.576{\hat{\sigma }}_{e,k}$, and the recursive variance estimate of [44] (forgetting factor 0.99, window 64); and R-ADFxMCC with $c_{\sigma }=2.5$, $N_{w}=64$, $\epsilon_{r}=10^{-6}$ and $\varsigma_{\min}=10^{-6}$. All augmented-diffusion methods use the same uniform admissible combination. The Swish-based algorithm [46] was not included because its complete update equations could not be reproduced from the available description. For CFxLMS, normalizing each acoustic path separately instead of by the total filtered-reference energy of the actuator amplifies weak cross-path regressors and, in our tests, caused divergence at every step size; the standard normalization is therefore used.

*Step-size selection.* Every method’s step size (and the penalty γ of SB-WCFxLMS) was selected by the same rule on five validation realizations (seeds 7500–7504), which are disjoint from all test seeds. For each reference spectrum, the candidate with the lowest median validation steady-state noise reduction (NR, defined below) among those with no divergent validation run was selected. The grids spanned more than two decades for every method except MDFxLMS, whose grid (0.05–0.8) covers its whole stable range. The selected values were interior points, except for CFxLMS on the single tone and the penalty of SB-WCFxLMS, where the smallest values were best; with the smallest penalty, SB-WCFxLMS essentially reduces to decentralized FxLMS. The selected values are listed in Table 4. As a secondary protocol, one common step for all four spectra was also selected for ADFxLMS, DADFxLMM and R-ADFxMCC. Throughout Section 5, λ denotes the step size of every algorithm; for the augmented-diffusion methods it is the step $\lambda_{k}$ of Equations (3) and (7), and the same value is used at all nodes.

*Code availability.* All results were produced by one simulator written in Python (version 3.11, Python Software Foundation, Wilmington, DE, USA), using fixed random seeds, and the timing results by a C benchmark compiled with GCC (version 13.3, Free Software Foundation, Boston, MA, USA). The code and the scripts that regenerate every figure and table are available as stated in the Data Availability Statement.

*Statistics.* Each test condition uses 30 paired realizations: all algorithms receive the same reference, paths, background noise, impulses and initial states. The acoustic performance measure is $\mathrm{NR}\left(n\right)=10\log_{10}\left[\sum_{k}{\left(d_{k}+y_{k}\right)}^{2}/\sum_{k}d_{k}^{2}\right]$. NR excludes the measurement noise, which is still present in the adaptation error $e_{k}\left(n\right)$. Curves are smoothed with a causal 200-sample energy window. The steady-state value of one realization is the mean of the smoothed NR (in dB) over the last 15% of the run. Results are summarized by the median and the interquartile range (IQR). Paired differences are reported as the median difference with a 95% percentile bootstrap confidence interval ($2\times 10^{4}$ resamples), the number of realizations in which R-ADFxMCC is better, and the two-sided Wilcoxon signed-rank *p*-value. A run is counted as divergent if its steady-state NR exceeds +20 dB.

### 5.2. Experiment 1: Mean-Convergence and EMSE Verification

Figure 1 compares the approximate mean recursion in Equation (16) with the Monte Carlo mean in Equation (13) under a fixed admissible block-row-stochastic combination matrix and $\lambda_{k}=0.4$. This experiment uses a synthetic linear version of the network, which satisfies (A1) exactly: L=J=64, single-tap secondary paths with the geometry of Section 5.1, a white Gaussian reference, an ideal solution at the origin, and a random unit-norm initial weight error (seed 20260808). The secondary-path length is increased from J=36 to J=64 so that no coupling is truncated: with J=36, the paths with delays above 35 samples are set to zero and only 70 of the 100 secondary paths remain, whereas with J=64 all 100 paths (maximum delay 42 samples) are retained. The synthetic model then contains every acoustic coupling, which (A1) requires. Sample-wise matrices in Equation (14) are estimated from $R_{\mathrm{est}}=500$ trials; a disjoint set of $R_{\mathrm{test}}=200$ trials produces the empirical mean weight-error norm. In panel (a), the measurement noise is white Gaussian with a standard deviation of 0.01 and $c_{\sigma }=0.6$, with a deliberately narrow kernel (mean gate value 0.52) chosen so that the gated statistics in Equation (14) differ markedly from the ungated ones. The curves differ by at most 0.002 dB during the initial transient (n≤10) and by 0.36 dB at n=599. Panel (b) uses BG impulses at all nodes ($p_{r}=0.06$, $\sigma_{\zeta }^{2}/\sigma_{\eta }^{2}=2\times 10^{4}$) and the operating value $c_{\sigma }=2.5$. The deviations are 0.025 dB and 0.33 dB, respectively. Repeating panel (a) with J=36 gives nearly the same agreement (0.004 dB during the transient and 0.35 dB at n=599), so the choice of *J* does not affect the conclusion. The small later separation is consistent with the independence approximation used to factor the gate, regressor, and weight error. Both curves remain bounded and decay, supporting the sufficient mean stability result, which holds under (A1)–(A3) and the filtered-x linearization.

The same synthetic network was used to test Equation (19). Starting from the ideal solution, 40 trials of 6000 samples were run, and the network EMSE $E[{\left(e_{k}-v_{k}\right)}^{2}]$ was averaged over the last 3000 samples and all nodes. Table 3 compares it with Equation (19), where $A_{k}$, $B_{k}$ and $C_{k}$ were evaluated from $4\times 10^{6}$ noise samples. Across all 18 cases, the expression exceeds the simulated network EMSE by 1.2–5.7 dB (median 3.8 dB). We attribute this offset mainly to the combination averaging and to the neglected disagreement between copies; it is of the same order for the MSE and correntropy updates. Because Equation (19) is a local approximation, this consistent overestimate is an empirical finding for this network, not a proven bound. The expression reproduces the effect of the impulses: at λ=0.4, the BG impulses raise the EMSE of ADFxLMS by 30.8 dB in simulation (30.8 dB predicted), but that of R-ADFxMCC ($c_{\sigma }=2.5$) by only 0.14 dB (0.16 dB predicted). The mean-square step limit $2B_{k}/C_{k}$ of the approximation equals 3.81 (Gaussian) and 3.52 (BG) for $c_{\sigma }=2.5$, so for this kernel factor, the mean condition $\lambda_{k}<2$ is the binding one. In all robust cases of Table 3, $B_{k}$ and $C_{k}$ were positive ($B_{k}=0.58$–0.80, $C_{k}=0.11$–0.44), so the sign conditions of Section 4.5 were met.

Finally, Proposition 1 was checked with 100 trials of 4000 samples (λ=0.4, $c_{\sigma }=2.5$) by measuring the steady-state mean of the a priori error relative to its standard error (*z*). With symmetric Gaussian noise, with or without an offset in the reference, |z|≤1.9 for both algorithms. With zero-mean skewed noise (centered exponential) and a zero-mean reference, the norm of the mean weight error of R-ADFxMCC stayed at the Monte Carlo level of the symmetric case, and its a priori error had a small offset (4.8% of its RMS value), as predicted by case (ii). When the reference was also given a nonzero mean, so that neither symmetry held, R-ADFxMCC acquired a clear offset (60% of the RMS a priori error, z=80), whereas ADFxLMS remained unbiased (|z|≤2.5).

### 5.3. Experiment 2: Localized Impulsive-Noise Robustness

**Figure 2:**
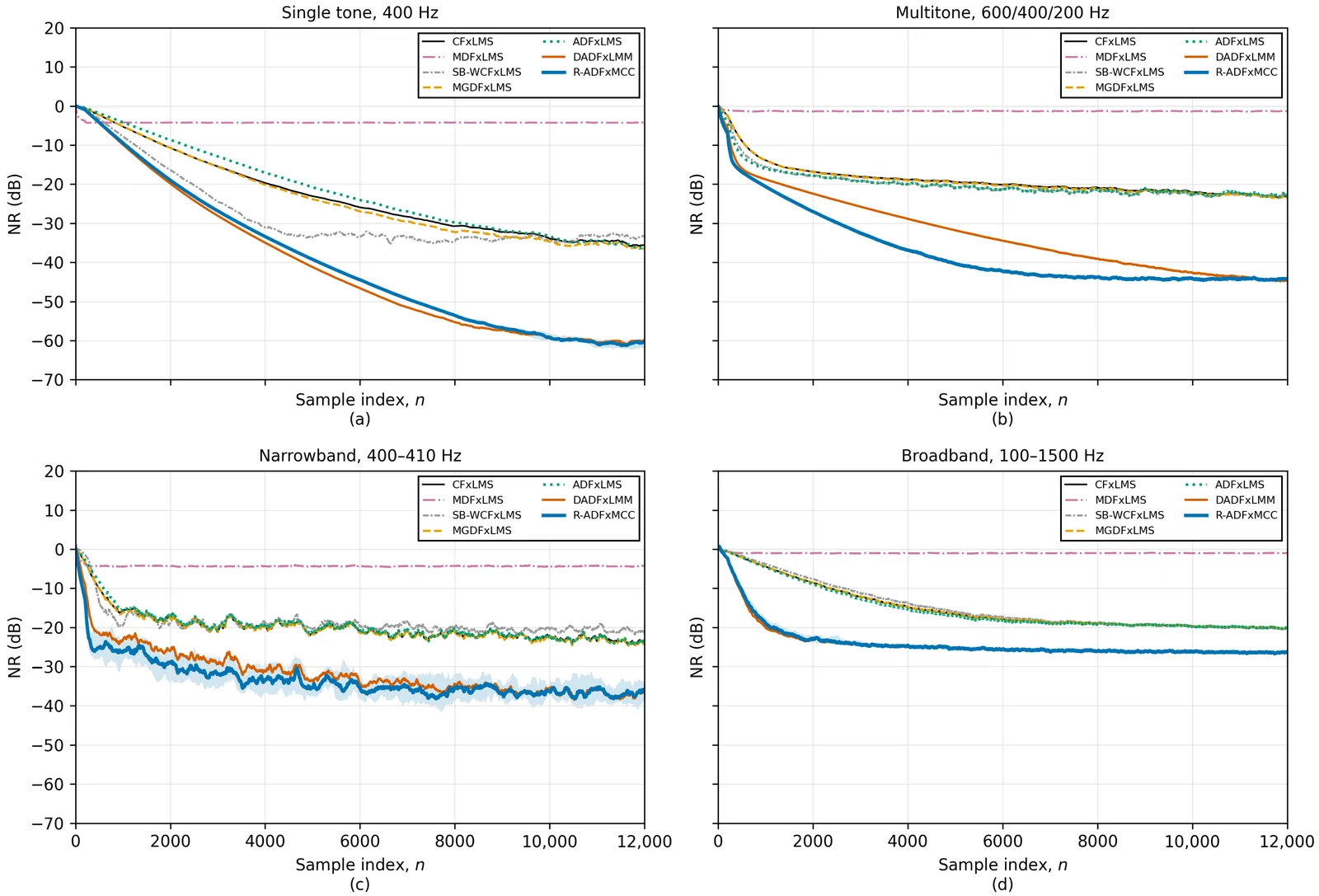
Localized BG-impulse comparison with every algorithm at its validation-selected step size: (**a**) single tone, (**b**) multitone, (**c**) narrowband, and (**d**) broadband. Lines are 30-realization medians; the R-ADFxMCC bands are interquartile ranges.

Localized BG impulses with $p_{r}=0.06$ are added at nodes {0,1,2} under single-tone, multitone, narrowband, and broadband references. All algorithms share identical signals, acoustic paths, impulses, and initial states within each realization. Figure 2 shows medians over 30 paired test realizations (seeds 8500–8529), with the IQR shown as a band for R-ADFxMCC. Table 4 lists the steady-state statistics, and Table 5 the paired comparisons with R-ADFxMCC.

With the validation-selected step sizes, none of the 840 test runs diverged. In particular, CFxLMS converges once it uses the standard normalization (Section 5.1). MDFxLMS remains limited to −1.02 to −4.27 dB at every step size, because it averages the unlike filters of neighboring nodes (the spatial-smoothing bias discussed in Section 1). The four mean-square-error methods (CFxLMS, SB-WCFxLMS, MGDFxLMS and ADFxLMS) reach nearly the same steady state: −35.63 to −33.45 dB for the single tone, −22.70 to −22.33 dB for the multitone, −23.33 to −20.48 dB for the narrowband, and −20.11 to −19.94 dB for the broadband. Under impulses, their floor is set by the impulse-driven misadjustment, which the small validation-selected step sizes reduce at the price of slow convergence (time to reach −10 dB, $t_{-10}$, of 1260–2314 samples on the single tone and 2228–2626 on the broadband reference). None of centralized control, gradient mixing or a weight constraint changes this, because none of them bounds the influence of an individual error sample.

R-ADFxMCC improves the median steady-state NR over ADFxLMS by 25.79, 21.84, 13.80 and 6.46 dB for the four references (wins 30/30, 30/30, 29/30, 30/30; Wilcoxon $p\leq 3.7\times 10^{-9}$). It also reaches −10 dB 1.6 to 4.7 times sooner ($t_{-10}$ of 1056, 228, 149 and 478 samples, against 2314, 356, 591 and 2228 for ADFxLMS).

The comparison with DADFxLMM answers whether the gain is specific to the correntropy formulation. It is not. The paired steady-state differences are 0.45, 0.36, −0.15 and 0.07 dB (95% confidence intervals [0.10, 0.80], [0.21, 0.48], [−0.35, 0.41] and [0.05, 0.08] dB). They are statistically significant for three references but no larger than half a decibel. The improvement over mean-square methods therefore comes from bounding the influence of impulses inside the augmented-diffusion update, which the M-estimate and the correntropy gate achieve to the same extent at their best step sizes. The two robust updates differ in their usable step-size range. At λ=0.8 and 1.2, ADFxLMS and DADFxLMM diverged in all 30 single-tone and all 30 narrowband realizations, whereas R-ADFxMCC diverged in none; its medians were −35.61 and −23.83 dB on the tone and −28.94 and −19.73 dB on the narrowband reference, i.e., stable but well above the floors reached at the selected steps. For the multitone reference, all three algorithms diverged in every realization at λ=1.2; at 0.8, none diverged, with medians of −7.86, −35.64 and −36.74 dB for ADFxLMS, DADFxLMM and R-ADFxMCC. This is consistent with Section 4.5: the smooth gate attenuates moderately large residuals as well as impulses, which lowers the effective step by the factor $B_{k}<1$, whereas the modified-Huber function passes every sub-threshold sample with unit weight. The selected R-ADFxMCC steps are also larger on the multitone and narrowband references, and R-ADFxMCC reaches −10 dB sooner there (228 versus 266 and 149 versus 212 samples). When one common step is used for all spectra (0.02, 0.1 and 0.1 for ADFxLMS, DADFxLMM and R-ADFxMCC), neither robust update dominates: the paired differences in favor of R-ADFxMCC are 0.87 dB [0.79, 0.90], −0.75 dB [−0.84, −0.72], 0.13 dB [0.09, 0.18], and −0.13 dB [−0.14, −0.12] for the four references (R-ADFxMCC better in 30/30, 0/30, 25/30, 1/30 realizations), and ADFxLMS loses up to 8.21 dB relative to its per-spectrum tuning.

### 5.4. Experiment 3: Sensitivity and Impulse Probability

**Figure 3:**
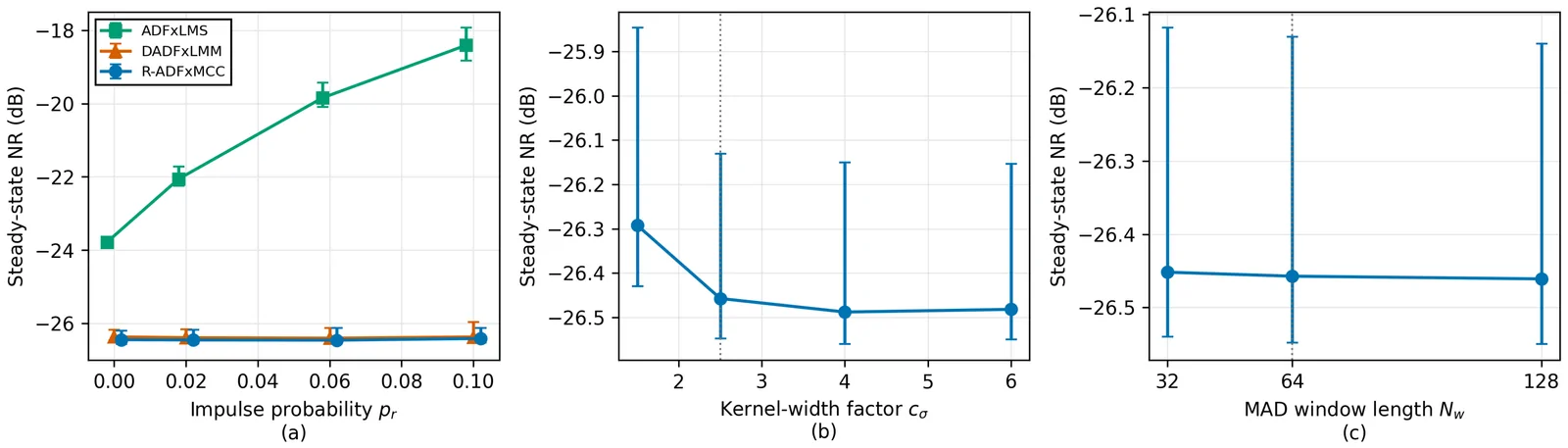
Broadband sensitivity over 30 paired realizations. Error bars are interquartile ranges: (**a**) impulse probability sweep for ADFxLMS, DADFxLMM and R-ADFxMCC; (**b**) R-ADFxMCC kernel-width-factor sweep at $p_{r}=0.06$ and $N_{w}=64$; and (**c**) MAD-window sweep at $p_{r}=0.06$ and $c_{\sigma }=2.5$. Step sizes are the validation-selected broadband values.

This experiment isolates the two robust-scale parameters and the impulse rate under the broadband reference. Thirty paired seeds, 8600–8629, are used; they do not overlap the four-spectrum or validation seeds. ADFxLMS, DADFxLMM and R-ADFxMCC use their validation-selected broadband step sizes (0.05, 0.4 and 0.4, respectively). The acoustic system, localized impulse nodes, variance ratio, and all other parameters remain as in Section 5.1. For the impulse probability sweep, $p_{r}\in \left\{0,0.02,0.06,0.10\right\}$, while $c_{\sigma }=2.5$ and $N_{w}=64$. The kernel-width sweep uses $c_{\sigma }\in \left\{1.5,2.5,4,6\right\}$, and the window sweep uses $N_{w}\in \left\{32,64,128\right\}$, both at $p_{r}=0.06$. Each point in Figure 3 is the median steady-state NR; the error bars span the interquartile range of the 30 realization-level values.

Without impulses and at the same step λ=0.4, ADFxLMS and R-ADFxMCC reach −26.47 and −26.45 dB (paired difference −0.02 dB, 95% CI [−0.05, −0.01]), so the performance penalty of the gate without impulses is negligible, although statistically detectable, in agreement with the limiting-case argument following Equation (10). This statement concerns noise reduction; the computational cost of the gate is reported in Section 3.5. At its validation-selected step, ADFxLMS degrades from −23.78 dB without impulses to −22.06, −19.84 and −18.41 dB at $p_{r}=0.02$, 0.06 and 0.10. R-ADFxMCC stays between −26.41 and −26.46 dB over the whole range, so its paired gain over ADFxLMS increases from 2.59 to 4.38, 6.38 and 7.79 dB. DADFxLMM behaves almost identically (between −26.37 and −26.40 dB), and its paired difference from R-ADFxMCC is between 0.05 and 0.07 dB at every impulse rate. Between $p_{r}=0.06$ and 0.10, R-ADFxMCC loses only 0.04 dB.

Across $c_{\sigma }=1.5$–6, the R-ADFxMCC median varies from −26.29 to −26.49 dB, a spread of 0.20 dB. The lowest factor is slightly worse (paired loss 0.15 dB relative to $c_{\sigma }=2.5$) because it attenuates more nonimpulsive residual samples. Across $N_{w}=32$, 64 and 128, the median changes by only 0.009 dB. Thus the default $\left(c_{\sigma },N_{w}\right)=\left(2.5,64\right)$ is not a narrow optimum. The step size has a larger effect: for λ=0.1, 0.2, 0.4 and 0.8, the broadband medians are −24.99, −26.09, −26.46 and −26.12 dB, and the smallest step does not fully converge within the run.

### 5.5. Experiment 4: α-Stable Noise, Reverberation and Secondary-Path Errors

**Figure 4:**
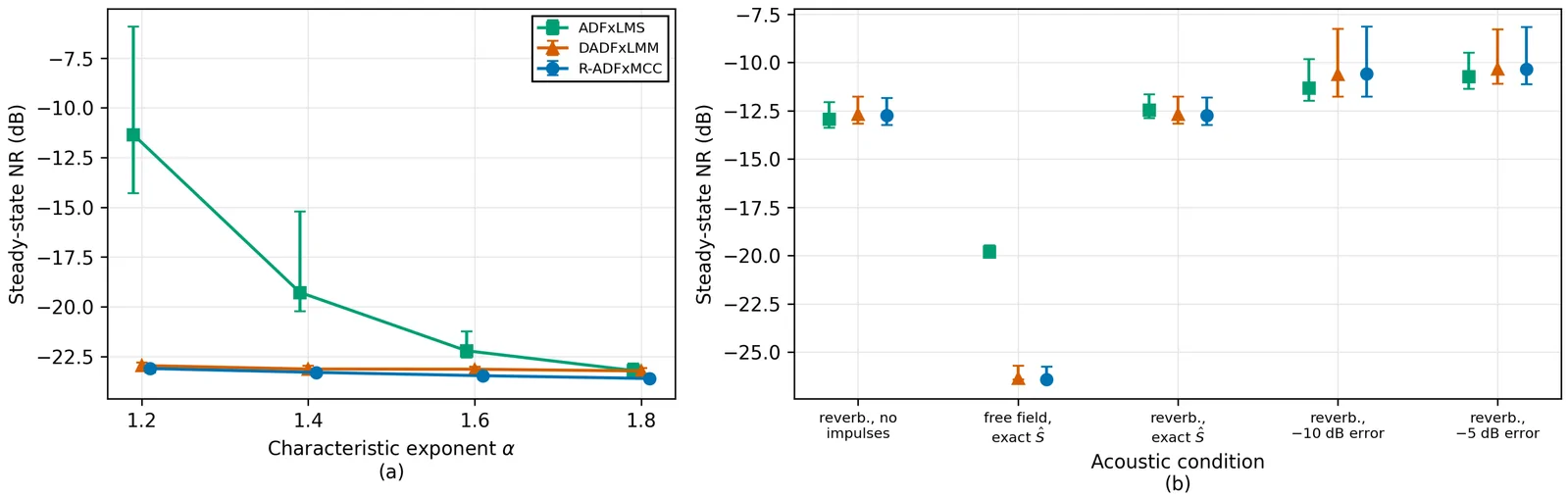
Broadband robustness tests over 30 paired realizations per condition (median and IQR): (**a**) SαS measurement noise at nodes {0,1,2} with scale five times the background standard deviation; (**b**) reverberant paths (direct-to-reverberant ratio 6 dB, $T_{60}=40$ ms) without impulses, the free field with BG impulses, and reverberant paths with BG impulses and exact, −10 dB and −5 dB misaligned secondary-path estimates. The last three conditions use runs of *N* = 36,000 samples; step sizes are the validation-selected broadband values.

*Symmetric α-stable noise.* SαS samples, generated by the Chambers–Mallows–Stuck method [53] with a scale of five times the background standard deviation, were added at nodes {0,1,2} for α∈{1.2,1.4,1.6,1.8} (seeds 8700–8729). Unlike BG noise, this contaminates every sample at these nodes. ADFxLMS degrades steadily as the tail becomes heavier, from −23.20 dB at α=1.8 to −11.34 dB at α=1.2. R-ADFxMCC stays between −23.11 and −23.61 dB and DADFxLMM between −22.95 and −23.23 dB (Figure 4a). The floor is about 3 dB higher than under BG impulses because the heavy-tailed component is present in every sample. R-ADFxMCC is consistently, although only slightly, better than DADFxLMM: the paired differences are 0.17, 0.21, 0.32 and 0.39 dB for α=1.2, 1.4, 1.6 and 1.8, with R-ADFxMCC better in 24/30, 28/30, 30/30, 30/30 realizations. This is consistent with the smooth gate down-weighting the moderately large samples that SαS noise produces continually, whereas the modified-Huber function passes every sub-threshold sample with unit weight.

*Reverberation and secondary-path errors.* Every primary and secondary path was given an exponentially decaying diffuse tail with a direct-to-reverberant ratio of 6 dB and $T_{60}=40$ ms (J=128, $J_{p}=192$), drawn independently for each realization (seeds 8800–8829). The secondary-path estimates were either exact or perturbed by white errors with −10 and −5 dB normalized misalignment, and localized BG impulses were present as in Experiment 2. With misalignment, none of the methods had converged within 12,000 samples, so all reverberant conditions are reported for runs of *N* = 36,000 samples (Figure 4b). The control filters remain L=64 taps long and cannot match the reverberant responses: without impulses, ADFxLMS, DADFxLMM and R-ADFxMCC reach −12.93, −12.66 and −12.75 dB. With impulses and exact estimates they reach −12.45, −12.66 and −12.73 dB. The robust methods are thus almost unaffected by the impulses, whereas ADFxLMS loses 0.48 dB, and R-ADFxMCC keeps a paired advantage of 0.33 dB [0.27, 0.38] over ADFxLMS. This advantage is much smaller than in the free field (Table 5), because the modeling error dominates the residual and the impulse-driven misadjustment is only a minor part of it. Misalignment raises the floor of all methods, to −11.33, −10.60 and −10.57 dB at −10 dB and to −10.74, −10.31 and −10.36 dB at −5 dB; no run diverged. In these conditions, R-ADFxMCC at its broadband step λ=0.4 is worse than ADFxLMS at λ=0.05 (paired −0.88 dB [−1.42, −0.31] and −0.52 dB [−0.81, −0.22]). The cause is the step size rather than the gate: at the same step 0.05, R-ADFxMCC reaches −11.34 and −10.97 dB, and its paired differences from ADFxLMS are 0.04 dB [−0.19, 0.28] and 0.18 dB [0.02, 0.29]. DADFxLMM behaves in the same way: at λ=0.05 it reaches −11.47 and −10.95 dB, compared with −10.60 and −10.31 dB at λ=0.4. With an inaccurate secondary-path model, both robust methods therefore need a smaller step than in the free field, and a step tuned in the free field should not be transferred unchanged (ADFxLMS was run only at its selected step). At λ=0.4, the paired median differences between R-ADFxMCC and DADFxLMM are at most 0.07 dB.

### 5.6. Reproducibility and Limitations

All performance comparisons use paired realizations: algorithms within a realization receive the same reference, primary and secondary paths, background noise, impulse locations and amplitudes, and initial states. The displayed time curves are causal 200-sample energy ratios, while every reported steady-state value is first averaged over the final 15% of one realization and then summarized across realizations. This order prevents a high-energy realization from dominating a pooled ratio. The source code records all seeds, the validation grids and the selected step sizes, and caches the smoothed NR curve of every run, so every number in this paper can be regenerated.

All step sizes were selected by one validation protocol, and the combination coefficients are uniform. The results therefore compare the local updates under equal conditions rather than jointly optimized networks. The steady-state criterion favors small steps; convergence times are reported alongside to make this trade-off visible.

The acoustic model is a two-dimensional free-field geometry with, as in Experiment 4, a synthetic exponentially decaying reverberant tail; measured room responses were not used. Actuator saturation, packet loss and clock asynchrony are outside the present scope. Experiment 4 showed that secondary-path errors call for smaller steps; combining the correntropy gate with online secondary-path modeling, as in [32], is a natural remedy that was not studied here. The mean recursion and the EMSE expression rely on the separation assumption (A3); the EMSE expression is a local approximation that treats each node in isolation and is not a proven bound on the network EMSE; for the network considered it overestimated the simulated value by 1.2–5.7 dB (median 3.8 dB). Validation with measured responses and real-time distributed hardware remains necessary before drawing deployment-level conclusions.

## 6. Conclusions

This paper presented R-ADFxMCC, a robust augmented-diffusion filtered-x algorithm for distributed multichannel ANC under impulsive noise. The method leaves the admissible augmented-diffusion combination structure and communication payload unchanged and introduces robustness only through a MAD-scaled correntropy gate in the local normalized update. The resulting influence is bounded and redescending. The analysis gave a combination-independent mean stability condition under the stated independence and linearization approximations, a symmetry-based proof of zero steady-state mean bias that does not require independence between the gate and the regressors, and a local steady-state EMSE approximation that explains why the correntropy gate removes the impulsive contribution to the misadjustment.

In 30-realization simulations, with every algorithm’s step size chosen by the same validation protocol, R-ADFxMCC improved the median steady-state noise reduction over ADFxLMS by 25.79, 21.84, 13.80 and 6.46 dB for single-tone, multitone, narrowband and broadband references under localized Bernoulli–Gaussian impulses. It showed a comparable margin over centralized FxLMS, the mixed-gradient algorithm and the weight-constrained decentralized algorithm, none of which bounds the influence of individual error samples. The augmented-diffusion M-estimate algorithm DADFxLMM reached essentially the same steady state (differences of at most 0.45 dB at the validation-selected step sizes), so the steady-state benefit is attributable to robustifying the augmented-diffusion update rather than to the correntropy criterion itself. The correntropy gate differs in three practical respects: on the single-tone and narrowband references, it remains stable at step sizes at which the M-estimate and mean-square updates diverge; it is slightly but consistently better under continuously heavy-tailed α-stable noise; and its smooth influence function admits the EMSE analysis given here. The gate adds no communication and, in the measured implementation, about 1955 ns per node and sample for the local update. Without impulses, its noise reduction penalty is negligible (0.02 dB at the same step size), and it is insensitive to $c_{\sigma }$ and $N_{w}$ over the ranges tested.

The work has limitations. The acoustic field is simulated, with a synthetic reverberant tail in one experiment. The EMSE expression is a local approximation that treats nodes in isolation; it overestimated the network EMSE in the case studied, but it is not a proven bound. A steady-state offset can arise when neither the residual nor the reference is sign-symmetric. With an inaccurate secondary-path model, a smaller step than in the free field was needed by both robust methods. Future work should extend the mean-square analysis to the network level, include adaptive or robust combination weights, and validate the method with measured room responses and real-time distributed hardware.

## Figures and Tables

**Table 1:** R-ADFxMCC at node *k*.

Step	Operation
0	Set $\xi_{k}\left(0\right)=0$; choose $\lambda_{k}$, $c_{\sigma }$, $N_{w}$, and $\epsilon_{r}$.
1	Acquire $e_{k}\left(n\right)$ and update $\sigma_{k}\left(n\right)$ using Equation (8).
2	Form ${\hat{r}}_{F,k}\left(n\right)$ and update $\varphi_{k}\left(n+1\right)$ using Equation (7).
3	Exchange intermediate augmented estimates with $p\in N_{k}$ and apply the fixed admissible combination in Equations (4) and (5).
4	Extract $w_{k}\left(n+1\right)=G_{k}^{k}\xi_{k}\left(n+1\right)$.

**Table 2:** Per-node implementation characteristics for one adaptation sample. The last column is the measured time of the local operations in Section 3.5 ($\delta_{k}=4$, $L=N_{w}=64$); it covers the local update only and excludes filtered-reference generation, control output filtering, I/O and communication.

Algorithm	Adaptive State	Principal Local Update	Additional Robust Work	Transmitted Intermediate Payload	Measured Time (ns)
MDFxLMS	*L*	Θ(L)	None	*L* scalars per neighbor	–
Normalized ADFxLMS	$\delta_{k}L$	$\Theta (\delta_{k}L)$	None	$\delta_{k}L$ scalars per exchange	835
DADFxLMM	$\delta_{k}L+N_{w}$	$\Theta (\delta_{k}L)$	One median, one square root	$\delta_{k}L$ scalars per exchange	1733
R-ADFxMCC	$\delta_{k}L+N_{w}$	$\Theta (\delta_{k}L)$	One exponential and two median evaluations	$\delta_{k}L$ scalars per exchange	2790

**Table 3:** Steady-state network EMSE (dB) of the synthetic network: simulation (40 trials) and the local approximation in Equation (19).

	Gaussian Noise			BG Impulses, $p_{r}$ = 0.06		
Algorithm, λ	0.1	0.4	1.0	0.1	0.4	1.0
ADFxLMS (simulation)	−56.60	−50.25	−45.64	−25.84	−19.49	−14.87
ADFxLMS (Equation (19))	−52.79	−46.03	−40.01	−21.98	−15.22	−9.20
R-ADFxMCC, $c_{\sigma }=1.5$ (simulation)	−58.52	−52.09	−47.72	−55.74	−51.89	−47.53
R-ADFxMCC, $c_{\sigma }=1.5$ (Equation (19))	−54.72	−48.57	−44.31	−54.55	−48.37	−44.04
R-ADFxMCC, $c_{\sigma }=2.5$ (simulation)	−57.51	−51.17	−46.69	−56.34	−51.03	−46.54
R-ADFxMCC, $c_{\sigma }=2.5$ (Equation (19))	−53.74	−47.35	−42.54	−53.62	−47.20	−42.29

**Table 4:** Experiment 2: validation-selected step sizes and median [IQR] steady-state NR (dB) over 30 test realizations. $t_{-10}$ is the median number of samples to reach −10 dB.

Algorithm	Single Tone	Multitone	Narrowband	Broadband
CFxLMS	−34.72 [−35.69, −33.46] λ=0.001; $t_{-10}=1854$	−22.48 [−22.78, −22.21] λ=0.01; $t_{-10}=573$	−23.18 [−24.04, −21.76] λ=0.005; $t_{-10}=494$	−20.04 [−20.33, −19.62] λ=0.02; $t_{-10}=2324$
MDFxLMS	−4.24 [−4.26, −4.23] λ=0.2; $t_{-10}$: –	−1.28 [−1.35, −1.22] λ=0.4; $t_{-10}$: –	−4.27 [−4.37, −4.09] λ=0.2; $t_{-10}$: –	−1.02 [−1.04, −0.99] λ=0.4; $t_{-10}$: –
SB-WCFxLMS	−33.45 [−35.71, −32.74] λ=0.0001, γ=0.1; $t_{-10}=1260$	−22.60 [−23.09, −22.13] λ=0.001, γ=0.1; $t_{-10}=418$	−20.48 [−21.24, −19.96] λ=0.001, γ=0.1; $t_{-10}=436$	−20.11 [−20.35, −19.75] λ=0.001, γ=0.1; $t_{-10}=2626$
MGDFxLMS	−35.63 [−36.92, −34.05] λ=0.001; $t_{-10}=1864$	−22.70 [−23.02, −22.41] λ=0.01; $t_{-10}=576$	−23.33 [−24.30, −21.92] λ=0.005; $t_{-10}=500$	−19.98 [−20.24, −19.56] λ=0.02; $t_{-10}=2318$
ADFxLMS	−34.97 [−35.85, −33.65] λ=0.002; $t_{-10}=2314$	−22.33 [−22.91, −22.05] λ=0.05; $t_{-10}=356$	−23.10 [−23.89, −21.73] λ=0.01; $t_{-10}=591$	−19.94 [−20.25, −19.53] λ=0.05; $t_{-10}=2228$
DADFxLMM	−60.28 [−61.00, −59.30] λ=0.005; $t_{-10}=1013$	−43.86 [−44.20, −43.66] λ=0.1; $t_{-10}=266$	−36.70 [−37.89, −35.33] λ=0.05; $t_{-10}=212$	−26.36 [−26.51, −26.20] λ=0.4; $t_{-10}=446$
R-ADFxMCC	−60.51 [−61.16, −59.78] λ=0.005; $t_{-10}=1056$	−44.23 [−44.37, −44.15] λ=0.2; $t_{-10}=228$	−36.94 [−38.41, −35.20] λ=0.1; $t_{-10}=149$	−26.43 [−26.59, −26.25] λ=0.4; $t_{-10}=478$

**Table 5:** Experiment 2. Paired steady-state improvement in R-ADFxMCC (dB, positive means R-ADFxMCC is better): median with 95% bootstrap confidence interval; wins out of 30 and Wilcoxon *p*-value.

Versus	Single Tone	Multitone	Narrowband	Broadband
CFxLMS	26.00 [25.47, 26.33] 30/30, $p=1.9\times 10^{-9}$	21.70 [21.48, 22.09] 30/30, $p=1.9\times 10^{-9}$	13.96 [12.84, 14.76] 29/30, $p=3.7\times 10^{-9}$	6.37 [6.16, 6.60] 30/30, $p=1.9\times 10^{-9}$
MDFxLMS	56.28 [55.94, 56.66] 30/30, $p=1.9\times 10^{-9}$	43.00 [42.85, 43.06] 30/30, $p=1.9\times 10^{-9}$	32.75 [31.47, 33.45] 30/30, $p=1.9\times 10^{-9}$	25.46 [25.33, 25.51] 30/30, $p=1.9\times 10^{-9}$
SB-WCFxLMS	26.75 [25.72, 27.47] 30/30, $p=1.9\times 10^{-9}$	21.52 [21.23, 22.09] 30/30, $p=1.9\times 10^{-9}$	15.81 [14.66, 17.27] 29/30, $p=3.7\times 10^{-9}$	6.36 [6.10, 6.58] 30/30, $p=1.9\times 10^{-9}$
MGDFxLMS	25.04 [24.28, 25.89] 30/30, $p=1.9\times 10^{-9}$	21.51 [21.25, 21.87] 30/30, $p=1.9\times 10^{-9}$	13.72 [12.66, 14.51] 29/30, $p=3.7\times 10^{-9}$	6.43 [6.20, 6.68] 30/30, $p=1.9\times 10^{-9}$
ADFxLMS	25.79 [25.09, 26.10] 30/30, $p=1.9\times 10^{-9}$	21.84 [21.37, 22.26] 30/30, $p=1.9\times 10^{-9}$	13.80 [12.63, 14.61] 29/30, $p=3.7\times 10^{-9}$	6.46 [6.18, 6.72] 30/30, $p=1.9\times 10^{-9}$
DADFxLMM	0.45 [0.10, 0.80] 21/30, p=0.009	0.36 [0.21, 0.48] 27/30, $p=6.9\times 10^{-6}$	−0.15 [−0.35, 0.41] 13/30, p=0.700	0.07 [0.05, 0.08] 27/30, $p=2.4\times 10^{-5}$

## Data Availability

The simulation code (Python 3.11 simulator and C timing benchmark compiled with GCC 13.3), the scripts that regenerate every figure and table from fixed random seeds, and the resulting data are available from the corresponding author upon reasonable request.

## Abbreviations

The following abbreviations are used in this manuscript:

ADFxLMS	Augmented-diffusion filtered-x least mean square
ANC	Active noise control
BG	Bernoulli–Gaussian
CFxLMS	Centralized filtered-x least mean square
DADFxLMM	Augmented-diffusion filtered-x least mean M-estimate algorithm of [43]
EMSE	Excess mean-square error
FxLMS	Filtered-x least mean square
FxMCC	Filtered-x maximum correntropy criterion
IQR	Interquartile range
MAD	Median absolute deviation
MCC	Maximum correntropy criterion
MDFxLMS	Conventional diffusion FxLMS baseline (Section 5.1)
MGDFxLMS	Mixed-gradient distributed filtered-x least mean square
MSE	Mean-square error
NR	Noise reduction
R-ADFxMCC	Robust augmented-diffusion filtered-x maximum correntropy criterion
SB-WCFxLMS	Self-boosted weight-constrained filtered-x least mean square
SαS	Symmetric α-stable
